# Motor sequence learning and learning to learn across the Alzheimer's disease continuum: Associations with biomarkers

**DOI:** 10.1177/13872877261485584

**Published:** 2026-09-08

**Authors:** Marit F. L. Ruitenberg, Paula D. Carmona Osorio, Jace B. King, John M. Hoffman, Kevin Duff, Vincent Koppelmans

**Affiliations:** 1 Department of Health, Medical and Neuropsychology, 4496 Leiden University, Leiden, the Netherlands; 2 Leiden Institute for Brain and Cognition, Leiden, the Netherlands; 3 Department of Radiology and Imaging Sciences, 219375 University of Utah, Salt Lake City, UT, USA; 4 172841 Department of Neurology, University of Utah, Salt Lake City, UT, USA; 5 Department of Neurology, 6684 Oregon Health & Science University, Portland, OR, USA; 6 Department of Psychiatry & Huntsman Mental Health Institute, University of Utah, Salt Lake City, UT, USA

**Keywords:** Alzheimer's disease, biomarkers, cognitive decline, mild cognitive impairment, motor learning, sequencing skill

## Abstract

**Background:**

While amnestic mild cognitive impairment (aMCI) and Alzheimer's disease (AD) are primarily associated with cognitive impairments, motor dysfunction has also consistently been observed. Less is known, however, about the acquisition of new motor sequences along the AD continuum.

**Objective:**

This study examined motor sequence learning in individuals with aMCI and AD dementia compared to cognitively normal individuals (CNI), and to what extent such learning is related to AD biomarkers.

**Methods:**

A total of 28 individuals with AD dementia, 33 with aMCI, and 52 CNI performed a computerized task that assessed general motor performance and motor sequence learning abilities. Participants also completed a neuropsychological test battery to assess their cognitive abilities. *APOE* genotyping was used to determine the presence of ɛ4 alleles, amyloid PET imaging to quantify amyloid deposition, and structural MRI scanning to obtain hippocampal volume.

**Results:**

While individuals with aMCI or AD dementia generally performed slower and less consistent than CNI, motor sequence learning occurred regardless of cognitive status. The rate of initial learning was slower in individuals with aMCI or AD dementia than in CNI. No significant associations between motor learning and AD biomarkers were observed. A combination of motor and cognitive measures best predicted amyloid burden, whereas a model with cognitive measures only best predicted hippocampal volume and *APOE* allele count.

**Conclusions:**

Overall, individuals with aMCI and AD dementia showed relatively preserved motor sequence learning abilities, despite some decrements in the initial rate of learning. Combining measures of motor and cognitive functioning may enhance the prediction of amyloid burden. Future studies should use more complex sequential movements and further explore the value of integrating motor measures in AD/aMCI assessment and monitoring.

## Introduction

While mild cognitive impairment (MCI) and Alzheimer's disease (AD) are primarily characterized by cognitive impairments, studies have demonstrated that individuals may also experience motor difficulties.^1,2^ People with MCI and AD generally show slower and less accurate performance on a wide range of fine and gross motor tests, and their performance can be used to accurately distinguish individuals with MCI and AD from cognitively normal individuals (CNI).^3–8^

Importantly, motor functioning does not only comprise an individual's ability to proficiently perform a variety of gross and fine motor behaviors—it also comprises the ability to learn such behaviors. Prior work on the impact of MCI and AD on the *acquisition* of movement sequences has yielded inconsistent results, with some studies reporting poorer motor sequence learning abilities compared to a control group and others suggesting intact abilities.^9–11^ The variable results in prior studies could be attributed to notable heterogeneity of study designs and experimental methodology used, as well as the focus on either AD or MCI in these studies. As such, it remains unclear how motor sequence learning is affected along the AD continuum. Furthermore, research in the motor domain has shown that people can *learn to learn* new skills.^12,13^ That is, repeated exposure to bouts of motor learning can lead to an overall improved ability to efficiently learn new motor skills. This concept of learning to learn, and how it might be disrupted, has received little attention in individuals with MCI and AD.

This study examined whether motor sequence learning and within-task learning to learn abilities are affected in individuals with amnestic MCI (aMCI; i.e., an intermediate state between normal ageing and AD dementia) and individuals with AD dementia compared to CNI. It was hypothesized that those with AD dementia, and to a smaller extent those with aMCI, would show poorer motor sequence learning and within-task learning to learn in addition to general motor deficits compared to CNI. Furthermore, associations between motor performance and learning abilities and three established biomarkers for AD were evaluated. These biomarkers comprised amyloid-β deposition obtained from positron emission tomography (PET) imaging, hippocampal volume obtained from structural magnetic resonance imaging (MRI) scans, and the number of *APOE* ɛ4 alleles obtained from blood samples.^14,15^ It was hypothesized that poorer motor performance and learning abilities would be related to higher biomarker load. Finally, this study sought to determine if motor sequence learning measures have the potential to enhance or outperform current neuropsychological assessment tools in predicting the presence of AD-associated biomarkers. It was hypothesized that a model comprising both motor and cognitive data would offer a superior prediction of biomarker load compared to a motor-only or a cognitive-only model. Findings from these analyses would be expected to have implications for clinical care and research in MCI and AD.

## Methods

### Participants

A total of 113 participants participated in the study, including 28 individuals with AD dementia, 33 individuals with aMCI, and 52 CNI. Table 1 provides an overview of their demographic and clinical characteristics. Participants in the present study overlapped with the sample reported in Duff et al.^16^ and Koppelmans et al.,^4,5^ who studied cognitive abilities and other motor abilities in aMCI and AD dementia, respectively.

**Table 1.** Demographic and clinical characteristics of the CNI, aMCI, and AD dementia groups.

Group:		CNI (*n* = 52)	aMCI (*n* = 33)	AD dementia (*n* = 28)	Group difference
***Demographics***					
Age (y)		74.0 ± 5.7	74.6 ± 6.0	75.5 ± 6.6	*F* = 0.79, *p* = 0.46, *η*^2^ = 0.01
Sex (M/F)		32F/20M	13F/20M	17F/11M	χ^2^=4.51, *p* = 0.104
Education (y)		16.7 ± 2.3	16.2 ± 2.7	15.1 ± 2.3	*F* = 3.89, *p* = 0.023, *η*^2^ = 0.07
WRAT (raw)		113.1 ± 8.3	109.5 ± 9.2	108.0 ± 8.7	*F* = 3.65, *p* = 0.03, *η*^2^ = 0.06
GDS (raw)		1.0 ± 1.3	1.6 ± 1.3	1.6 ± 1.5	*F* = 2.29, *p* = 0.106, *η*^2^ = 0.04
MMSE (raw)		29.0 ± 1.1	26.3 ± 1.9	22.9 ± 2.6	*F* = 101, *p* < 0.001, *η*^2^ = 0.65
CDR Global		0.0 ± 0	0.5 ± 0	0.7 ± 0.2	*F* = 370, *p* < 0.001, *η*^2^ = 0.88
WMS-R (raw)		12.4 ± 3.6	3.2 ± 3.1	1.3 ± 1.6	*F* = 152, *p* < 0.001, *η*^2^ = 0.73
***Cognitive tests***					
HVLT-R (total recall, raw)		26.90 ± 5.32	16.52 ± 4.73	11.39 ± 3.98	*F* = 105.2, *p* < 0.001, *η*^2^ = 0.66
HVLT-R (delayed recall, raw		9.79 ± 1.92	3.15 ± 3.34	0.61 ± 1.23	*F* = 169.8, *p* < 0.001, *η*^2^ = 0.76
BVMT-R (total recall, raw)		25.38 ± 5.31	12.42 ± 5.65	6.96 ± 3.09	*F* = 144.9, *p* < 0.001, *η*^2^ = 0.73
BVMT-R (delayed recall, raw)		9.77 ± 1.99	4.30 ± 3.29	1 ± 1.22	*F* = 143.4, *p* < 0.001, *η*^2^ = 0.72
SDMT (raw)		46.2 ± 8.32	38.2 ± 7.22	27.7 ± 8.40	*F* = 48.61, *p* < 0.001, *η*^2^ = 0.47
RBANS (standard scores^a^)					
	IMI	111.24 ± 11.69	79.46 ± 14.80	62.77 ± 11.70	*F* = 144.8, *p* < 0.001, *η*^2^ = 0.73
	DMI	112.88 ± 12.54	67.34 ± 22.29	51.29 ± 11.47	*F* = 160.8, *p* < 0.001, *η*^2^ = 0.75
	VCI	110.45 ± 11.62	98.46 ± 14.61	92.92 ± 13.25	*F* = 18.55, *p* < 0.001, *η*^2^ = 0.26
	LI	107.35 ± 10.30	91.34 ± 10.99	80.62 ± 14.56	*F* = 54.1, *p* < 0.001, *η*^2^ = 0.48
	AI	108.74 ± 14.39	96.78 ± 16.56	86.74 ± 14.38	*F* = 19.8, *p* < 0.001, *η*^2^ = 0.27
***AD biomarkers***					
Amyloid SUVR		0.3 ± 2.4	5.0 ± 3.3	6.3 ± 3.5	*F* = 44.4, *p* < 0.001, *η*^2^ = 0.45
Hippocampal volume		4.3 ± 0.6	3.7 ± 0.8	3.2 ± 0.8	*F* = 23.9, *p* < 0.001, *η*^2^ = 0.31
No. of *APOE* ɛ4 alleles^b^					Fisher's *p* < 0.001
	0	39	8	9	
	1	12	17	12	
	2	1	7	7	
Post hoc amyloid status^b^					
	Positive	8	24	25	
	Negative	39	5	2	
	Uncertain	5	3	1	

WRAT: Wide Range Achievement Test-4 reading subtest; GDS: Geriatric Depression Scale; MMSE: Mini-Mental State Examination; CDR: Clinical Dementia Rating scale; WMS-R: the Wechsler Memory Scale-Revised Logical Memory II Paragraph A; HVLT-R: Hopkins Verbal Learning Test – Revised; BVMT-R: Brief Visuospatial Memory Test – Revised ; SDMT: Symbol Digit Modality Test; RBANS: Repeatable Battery for the Assessment of Neuropsychological Status; IMI: Immediate Memory Index; DMI: Delayed Memory Index; VCI: Visuospatial/Constructional Index; LI: Language Index; AI: Attention Index; SUVR: ^18^F-Flutemetamol PET scan global composite standardized uptake value ratio.

^a^ Standard score with M = 110, SD = 15.

^b^ Missing for 1 participant.

Inclusion criteria were (a) age 65 years or older and (b) availability of a knowledgeable collateral source to comment on their cognition and daily functioning. Exclusion criteria comprised (a) medical comorbidities likely to affect cognition (i.e., neurological conditions, current severe depression, substance abuse, and major psychiatric conditions); (b) inability to complete MRI or PET imaging; (c) inability to complete cognitive assessments due to inadequate vision, hearing, or manual dexterity; (d) being enrolled in a clinical drug trial related to anti-amyloid agents; (e) elevated depression as indicated by a score of greater than 5 on the 15-item Geriatric Depression Scale; and (f) severe dementia as indicated by a Clinical Dementia Rating Scale (CDR) score of 2 or greater or a Mini-Mental State Examination (MMSE) score of less than 20.

CNI were recruited from the community. The majority of aMCI and AD dementia participants were recruited from the University of Utah cognitive disorders’ clinic. A minority of aMCI subjects came from the community sample who met criteria after cognitive evaluation. The study was approved by the Institutional Review Board of the University of Utah. All CNI and those with aMCI provided written informed consent, whereas individuals with AD dementia provided assent while a legally authorized representative provided consent on their behalf.

Classification of the clinical groups was performed by a neuropsychologist and based on the Alzheimer's Disease Neuroimaging Initiative (ADNI)^17^ classification battery, which comprises the MMSE,^18^ the CDR,^19^ and the Wechsler Memory Scale-Revised (WMS-R)^20^ Logical Memory II Paragraph A. Participants were categorized into the CNI group if they had an MMSE 24–30, a CDR of 0, and a WMS-R score of ≥9 with ≥16 years of education, ≥5 with 8–15 years of education, or ≥3 with 0–7 years of education. Participants were categorized into the aMCI group if they had an MMSE 24–30, a CDR of 0.5, and a WMS-R score of ≤8 with ≥16 years of education, ≤4 with 8–15 years of education, or ≤2 with 0–7 years of education. Participants were categorized into the AD dementia group if they had an MMSE 20–26, a CDR of 0.5 or 1, and a WMS-R score of ≤8 with ≥16 years of education, ≤4 with 8–15 years of education, or ≤2 with 0–7 years of education. In cases where participants met criteria for more than one diagnostic group due to overlapping ADNI score ranges, the degree of functional impairment as reflected in the CDR score was prioritized to guide classification.

To evaluate the effectiveness of the ADNI criteria in accurately categorizing participants in these groups, post hoc amyloid status classification was performed based on both visual read of ^18^F-Flutemetamol-PET scans and quantitative metrics (composite SUVR and Z-score). Participants were considered amyloid-positive when visual and quantitative findings indicated elevated amyloid burden, and as amyloid-negative when both measures supported the absence of significant amyloid deposition. Some participants were classified as “uncertain” because visual inspection revealed features suggestive of localized neuritic amyloid plaques, while quantitative measures remained within two standard deviations of the normative reference range. This category was introduced to avoid assigning a definitive positive or negative classification in cases with discordant visual and quantitative findings. The distribution of amyloid positive/negative/uncertain participants per group is shown in Table 1.

### Motor sequence learning task

As illustrated in Figure 1, participants were shown a series of four different colored arrows on a screen, with each arrow having a specific color, direction, and location. They were provided with a gaming controller containing four buttons, each representing one of the four possible arrows. Participants were instructed to press the corresponding buttons on the game controller to match the arrow displayed on the screen.

**Figure 1.**
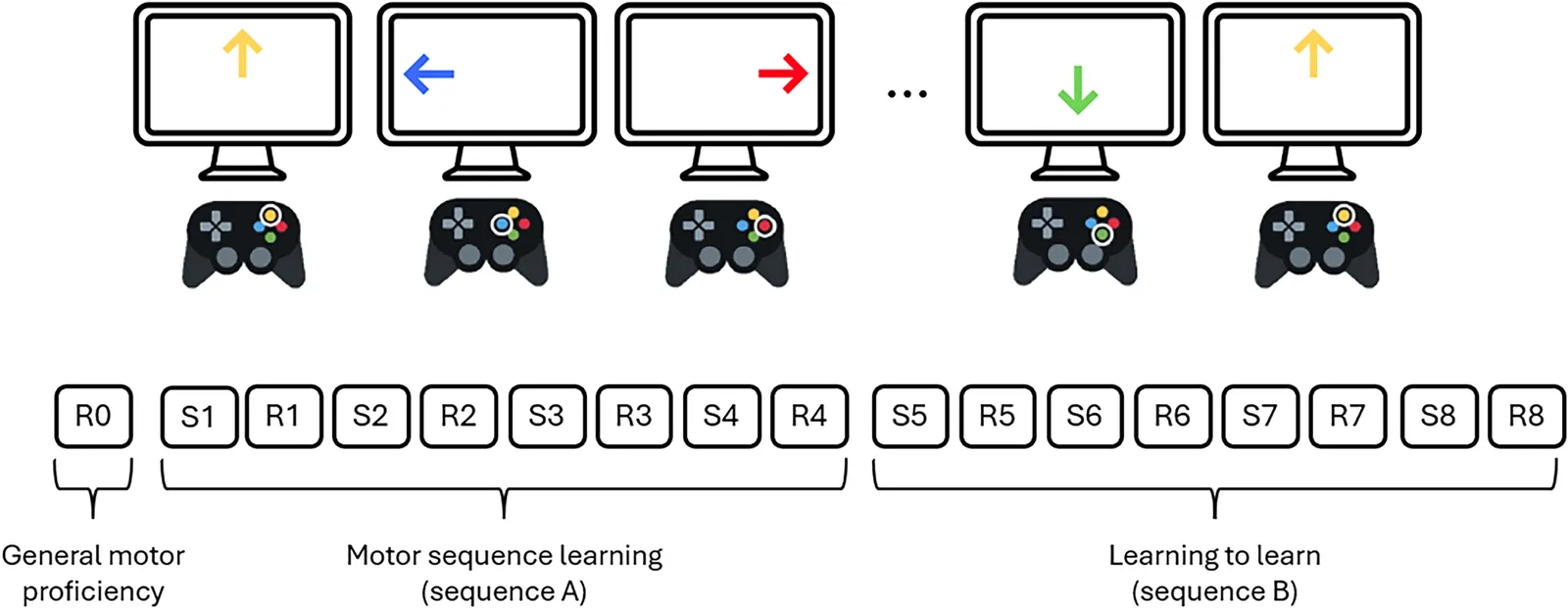
Stimulus presentation involved the display of arrows that each corresponded to one of four response buttons. In alternating blocks of 32 stimuli each, the arrows either followed a pseudorandom order (R) or involved the repetition of a 4-element sequence (S).

The task started with a block in which stimuli were presented in a pseudorandom order (i.e., no direct repetitions) to evaluate participants’ general motor proficiency. Then, eight sets of alternating random and sequence blocks were presented—each random block encompassed 32 pseudorandom trials, whereas each sequence block encompassed eight repetitions of the same 4-element sequence. The relatively short 4-element sequence was selected to allow all participants, including those with AD dementia, to pick up on the regularity of the responses. Two different sequences were displayed during the task: one in the sequence blocks of the first half of the task (cf. motor sequence learning), and another in the sequence blocks of the latter half of the task (cf. within-task learning to learn).

During task performance, the reaction time (RT; in seconds) and accuracy of the response were logged for each trial. Data preprocessing subsequently involved the exclusion of incorrect responses (7.1%) and outliers (1.9%). Outliers were defined as trials on which the RT deviated more than 3 standard deviations from the mean per block per participant. For each individual, the mean RT and coefficient of variation (CoV; mean RT divided by standard deviation) per block were subsequently determined. In addition, the rate of learning was quantified by calculating the linear slope coefficient across RT differences between pairs of adjacent random-sequence blocks across the task (done separately for the first and second half of the task).

### Biomarkers

Three well-established AD biomarkers were cross-correlated with motor sequence learning performance, namely whole brain amyloid-β deposition, hippocampal volume, and *APOE* ɛ4 allele status. The data collection procedures used in this study have been described in detail elsewhere.^4,5,16^ In brief, ^18^F-Flutemetamol PET imaging of the brain was used to estimate amyloid-β neuritic plaque density, expressed as a composite standardized uptake value ratio (SUVR) in the cerebral cortex and normalized to the pons. Furthermore, FreeSurfer processing of structural T1-weighted MRI scans was used to obtain hippocampal volumes, which were expressed as the proportion of the estimated total intracranial volume. Finally, *APOE* genotyping based on whole blood samples was used to classify participants into three groups of having either 0, 1, or 2 *APOE* ɛ4 alleles.

### Cognitive assessments

The neuropsychological test battery included four cognitive assessments. First, the Repeatable Battery for the Assessment of Neuropsychological Status (RBANS)^21^ is a brief neuropsychological testing battery that includes 12 subtests used to calculate index scores on five different domains: immediate memory, visuospatial/constructional abilities, attention, language, and delayed memory (jointly resulting in an overall score). Using normative data from the test manual, these index scores are converted to age-corrected standard scores, with higher scores reflecting better cognitive performance. The Hopkins Verbal Learning Test-Revised (HVLT-R)^22^ is a verbal learning and memory task. It consists of three free recall learning trials in which a list of 12 words is read to participants and they are asked to repeat them immediately. After a delay of 20–25 min, participants are asked to recall the list of words they were presented earlier. Scoring comprises the number of correctly recalled words, resulting in scores ranging from 0 to 36 for immediate recall (12 words across 3 trials) and 0 to 12 for delayed recall, with higher scores indicating better verbal memory performance. The Brief Visuospatial Memory Test-Revised (BVMT-R)^23^ is a visuospatial memory task that involves six geometric designs presented on six locations on a card. Participants study the card and copy the designs in their respective locations across three trials. Scoring comprises the number of correct designs and locations, resulting in scores ranging from 0 to 36 for immediate recall (6 designs and 6 locations across 3 trials) and 0 to 12 for delayed recall, with higher scores indicating better visuospatial memory performance. Finally, the Symbol Digit Modalities Test (SDMT)^24^ was used to assess sustained attention and psychomotor speed. It involves the substitution of digits for abstract symbols using a reference key. Participants are asked to correctly pair as many symbols and numbers as possible in 90 s. Scoring was based on the number of correct substitutions within the 90-s interval; higher scores represent better information processing speed.

### Statistical analysis

All statistical analyses were conducted using R version 4.3.1.^25^ The alpha threshold was set at 0.05. Group differences in demographic variables, symptoms of depression, and general motor proficiency data were analyzed using one-way analyses of variance (ANOVAs) for continuous variables (e.g., age, years of education) and Chi^2^ or Fisher's exact test for categorical variables (e.g., sex, number of *APOE* ɛ4 alleles). For cognitive test data, separate ANCOVAs were performed for each test, treating group as a between-subjects factor and adjusting for education as a covariate. Group differences in general motor proficiency were assessed using ANOVAs on mean RT and CoV for the first random block, with group as a between-subjects factor. White's heteroskedasticity-consistent standard errors were used where homogeneity of variances was violated. Post-hoc pairwise t-tests with Bonferroni correction were used to analyze between-group differences in the case of significant main effects.

RT and CoV for the motor sequence learning and within-task learning to learn sequences were compared between groups using separate mixed ANOVAs with group as a between-subjects factor and learning type, block type, and block number as within-subjects factors. To compare group differences in sequence learning rates, mixed ANOVAs were employed on learning rate with group as a between-subjects factor and learning type and block type as within-subjects factors. Post-hoc analyses of significant effects were performed using Tukey's method.

To examine associations between motor measures and hippocampal volume or amyloid burden, linear regressions were used for each biomarker with the motor variables as predictors. For *APOE* ɛ4 allele count, Poisson regressions were used instead. Age and sex were included as covariates in all regression models.

Finally, it was evaluated whether using motor measures could enhance the prediction of AD biomarker presence. First, principal component analyses (PCA; ‘prcomp’ function from the ‘stats’ package) were applied to the sets of motor and cognitive measures for dimensionality reduction; Supplemental Figure 1 shows the individual measures that were included. For both the motor and cognitive datasets, principal components with eigenvalues greater than one were retained, following the Kaiser criterion, ensuring that those components explained the most variance. For missing values in the cognitive variables (n = 3 missing RBANS scores), a simple imputation method was employed, replacing the missing values with the mean of each group for the respective cognitive test. Next, three linear regressions were run for each biomarker: a motor-only model, a cognitive-only model, and a combined model. Finally, model performance in predicting each biomarker was compared using Akaike Information Criterion (‘AIC’ function from the ‘stats’ package). The AIC is a metric used for evaluating and comparing statistical models by balancing goodness-of-fit against model complexity. It estimates the relative quality of models, where a lower AIC value indicates a better model.^26^

## Results

### General motor proficiency

Performance in the first random block was evaluated to examine differences in general motor proficiency. Results of two ANOVAs on RT and CoV with Group (3) as between-subject variable showed significant differences among groups, for both RT, *F*(2,110) = 20.13, *p* < 0.001, *η^2^* = 0.27, and for CoV, *F*(2,110) = 14.27, *p* < 0.001, *η* *^2^* = 0.21 (see Figure 2). Post-hoc analyses revealed that participants in the AD dementia group displayed significantly longer RTs compared to both those in the CNI (*p* < 0.001) and aMCI groups (*p* < 0.001). However, RTs did not differ between the CNI and aMCI groups (*p* = 0.49). Furthermore, the AD dementia group showed significantly larger CoV than both the CNI (*p* < 0.001) and the aMCI groups (*p* = 0.033). The latter in turn exhibited more variability than the CNI group (*p* = 0.033).

**Figure 2.**
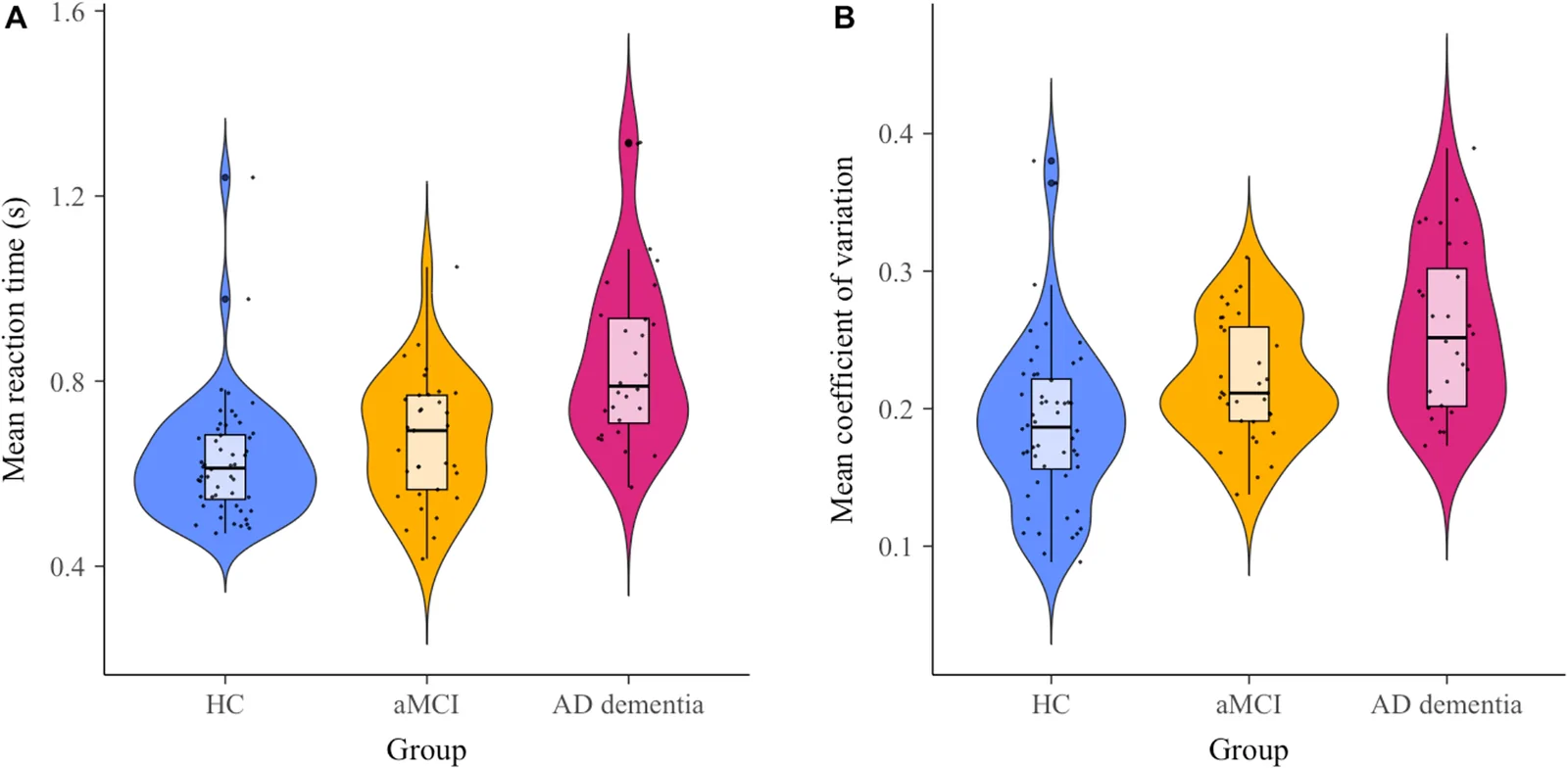
Mean reaction time (A) and mean coefficient of variation (B) in the first random block as a function of group.

### Motor sequence learning

Results of an ANOVA on RT with learning type (2; sequence learning vs. within-task learning to learn), block set (4), and block type (2; sequence vs. random) demonstrated a significant main effect of group, *F*(2,1760) = 11.56, *p* < 0.001, *η^2^* = 0.22. Post hoc pairwise t-tests showed that RTs were shortest in the CNI group, followed by the aMCI group, while the AD dementia group exhibited the longest RTs (*p*s < 0.001). Additionally, RTs were shorter in sequence blocks compared to random blocks, *F*(2,1760) = 23.33, *p* < 0.001, *η^2^* = 0.14. No other significant main or interaction effects were observed (all *p*s > 0.05). Results of a similar ANOVA on CoV also showed significant main effects of group, *F*(2,1760) = 13.20, *p* < 0.001, *η^2^* = 0.06, and block type, *F*(2,1760) = 4.56, *p* = 0.033; *η^2^* = 0.05. Post hoc tests showed that CoV was largest in the AD dementia group, followed by the aMCI group, whereas the CNI group showed the smallest CoV (*ps* < 0.001). No other significant main or interaction effects were observed (all *p*s > 0.143).

When examining learning rates, results of a mixed ANOVA on slope coefficients with group (3) as between-subject factor and learning type (2) as within-subject factor revealed no main effects or interactions (*p*s > 0.06). Given the relatively simple nature of the 4-element sequences, it was conceivable that performance may have rapidly improved and then stabilized (i.e., showed a ceiling effect) within the first blocks of each half of the task, such that any performance differences were obscured when considering the task in its entirety. To statistically evaluate this exploratory hypothesis, an exponential decay function was used to determine the decay constant for each participant as a measure of initial learning rate across trials in the first sequence blocks. Next, an ANOVA on these decay constants with learning type (2) as a within-subject variable and group (3) as a between-subjects variable was performed. As illustrated in Figure 3, results revealed a significant main effect of group, *F*(2,220) = 5.93, *p* = 0.003, *η^2^* = 0.05. Post-hoc Tukey tests indicated that the CNI group exhibited a significantly faster exponential decay of RTs compared to the aMCI (*p* = 0.034) and the AD dementia groups (*p* = 0.005). No significant difference was observed between the aMCI and AD dementia groups (*p* = 0.780).

**Figure 3.**
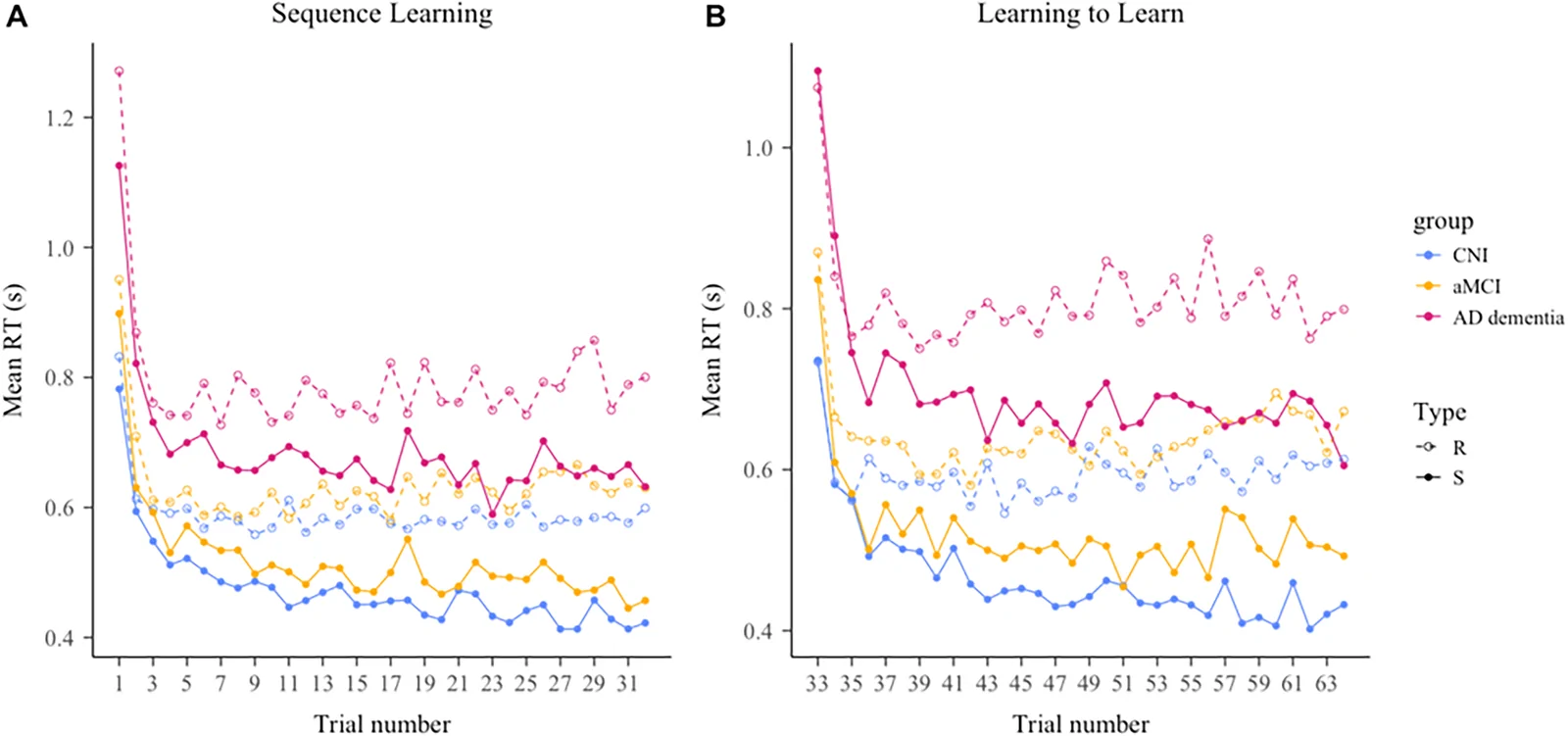
Mean RT across trials in the first sequence block for the first half (A) and second half (B) of the task as a function of group.

### Associations with biomarkers

Table 2 presents an overview of the associations between each motor measure and the AD biomarkers. Results showed that longer RTs and larger CoVs (both reflecting poorer general motor proficiency) were associated with higher amyloid deposition and smaller hippocampal volume (see Figure 4). No significant associations between sequence learning rates and biomarkers were observed.

**Figure 4.**
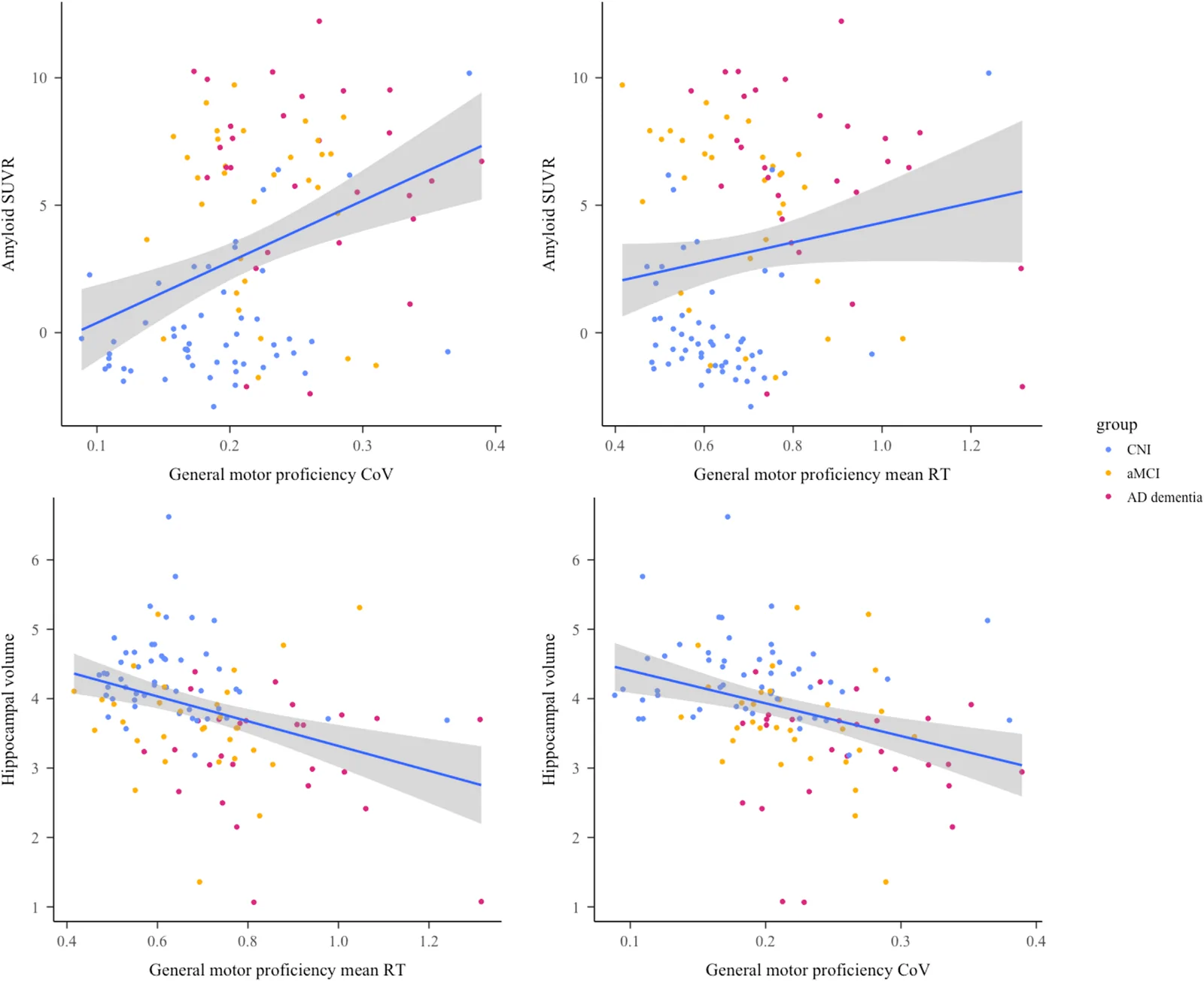
Scatter plots showing significant associations between motor measures and AD biomarkers. Note that while individual observations are color-coded for group, analyses were collapsed across groups.

**Table 2.** Results of regression analyses evaluating the associations between AD biomarkers and motor performance measures.

Biomarker	General motor proficiency		Learning rates (slope across blocks)		Initial learning rates (exponential decay constant)	
	**RT**	**CoV**	**Sequence learning**	**Learning to learn**	**B1 sequence learning**	**B1 learning to learn**
Amyloid SUVR	**β = 4.93, *p*** **=** **0.037**	**β = 24.99, *p*** **<** **0.001**	**β = −**2.35, *p* = 0.88	**β = −**27.12*, p* = 0.068	**β = −**69.22, *p* = 0.14	**β = −**19.30, *p* = 0.56
Hippocampal volume	**β = −1.26, *p*** **=** **0.006**	**β = −4.12, *p*** **<** **0.001**	**β = **3.32, *p* = 0.28	**β = −**0.59, *p* = 0.84	**β = **16.49, *p* = 0.077	**β = **4.01, *p* = 0.53
*APOE* ɛ4 alleles	**β = **0.41, *p* = 0.35	**β = **1.111, *p* = 0.36	**β = **0.86, *p* = 0.78	**β = −**0.52, *p* = 0.86	**β = −**0.41, *p* = 0.96	**β = **0.55, *p* = 0.93

### Predicting AD biomarker burden

Results of the PCAs revealed three motor components and one cognitive component (see Supplemental Figure 1), which were used as predictor variables for the subsequent biomarker prediction models. For hippocampal volume, results showed that the cognitive model provided the best fit, *F*(3,105) = 22.46, *p* < 0.001, *R^2^* = 0.373, AIC = 230.03. For amyloid burden, the combined model comprising both motor and cognitive components demonstrated providing the best fit, *F*(6,105) = 14.83, *p* < 0.001, *R^2^* = 0.459, AIC = 576.20. The *APOE* ɛ4 models, analyzed with Poisson regression, showed that the cognitive model provided the best fit (AIC = 292.47, residual deviance = 20.43, df = 108). To compare the fit of the models across all biomarkers, the AIC values for each model are summarized in Table 3.

**Table 3.** AIC values for the cognitive, motor, and combined prediction models as a function of AD biomarker.

Biomarker	Cognitive model	Motor model	Combined model
Amyloid SUVR	580.74	628.77	**576** **.** **20**
Hippocampal volume	**230**.**03**	260.89	234.98
*APOE* ɛ4 alleles	**292**.**47**	303.98	296.34

The models with the best fit are presented in bold.

## Discussion

The present study examined how motor sequence learning and within-task learning to learn are affected along the AD continuum. Motor responses of individuals with aMCI and AD dementia generally were slower and more variable compared to CNI, which is in line with previous observations during different motor tasks in the present sample^4,5^ as well as independent samples.^7^ These decrements in motor performance are thought to be due to AD-related neuropathological,^27–29^ which is supported by current observations that poorer general motor proficiency was associated with higher amyloid burden and smaller hippocampal volume.

With regards to the learning of new movement sequences, the present findings indicate that aMCI and AD dementia do not affect individuals’ ability to acquire such sequences nor their ability to learn to learn. This suggests that motor learning abilities for short, deterministic sequences are relatively preserved in aMCI and AD dementia, which could have implications for the design of rehabilitation programs. Specifically, for individuals with aMCI or AD dementia in physical rehabilitation settings, clinicians may focus on motor learning techniques without needing to modify such interventions based on cognitive status—although there are some important nuances that should be highlighted. For instance, this implication may only apply to relatively simple sequences but not more complex ones (see below). Such a nuanced view ties in with observations from reviews on motor learning in MCI^10,11^ and AD,^9^ which suggest that mixed results across prior studies may be due to differences in learning paradigms and study designs. For example, individuals with MCI showed poorer learning of an implicit, probabilistic sequence,^30^ while this was not the case in studies using a fixed sequence (e.g., De Wit et al.^31^ and the present study). Further investigations should work towards a more comprehensive understanding of the specific motor learning conditions that are (not) affected by cognitive impairments.

Furthermore, exploratory analyses revealed that individuals with aMCI and AD dementia learn at a slower rate than CNI during the initial stage of motor sequence learning. Such early learning of movement sequences typically requires explicit cognitive processes and has previously been associated with strong hippocampal involvement.^32,33^ Specifically, recent work suggests that the hippocampus plays a key role in the temporal ordering of movement sequences,^34,35^ which is particularly important during initial stages of learning where participants are picking up on the temporal regularity in the task. It is conceivable that the slower initial learning rates observed in aMCI/AD dementia individuals could be explained by the corresponding group differences in hippocampal volume in the present study, which are in line with previous reports that hippocampal atrophy is linked to AD progression and differentiates between CNI and aMCI/AD individuals.^36,37^ However, as we did not observe significant brain-behavior associations for any of the learning rates, our hippocampal volume measure may be more reflective of general neurodegeneration (including that of cortical motor areas and the cerebellum) rather than just hippocampal atrophy.

When evaluating the associations of motor sequence learning and motor performance with AD biomarkers, results showed that higher amyloid burden and smaller hippocampal volumes were associated with poorer general motor performance. These results align with prior work showing that finger tapping speed and variability were related to hippocampal volume in a sample comprising CNI, aMCI, and AD dementia participants that largely overlaps with the present sample.^4^ In cognitively unimpaired adults, presence of amyloid pathology has also been associated with measures of motor performance, such as increased reaction time variability^38^ and slower gait speed.^39^ However, as aforementioned, we did not observe associations with any of our learning indices. Taken together, these findings suggest that as AD neuropathology progresses, several types of motor function may be impacted – yet this impact seems to mostly pertain to motor *performance* rather than motor *learning*. The lack of associations between motor measures and *APOE* ɛ4 may relate to genetics being a non-modifiable marker for risk of developing the disease,^40^ which in turn may interact with environmental and lifestyle factors,^41^ rather than a reflection of disease pathology.

Lastly, the present findings suggest that including motor measures alongside cognitive measures could improve the prediction of amyloid burden, but not hippocampal volume or number of *APOE* ɛ4 alleles. While many studies have established a link between higher amyloid deposition and motor performance, to our best knowledge, only one other study has demonstrated the potential of a motor learning task to predict amyloid burden. Schaefer et al.^8^ demonstrated that including performance on a simple timed upper-limb coordination task improved model prediction of amyloid burden in individuals with aMCI. The present study is the first to show that such additional predictive value also extends to the AD continuum. With regard to the clinical significance, we acknowledge that the additional explained variance is modest. While the present finding demonstrates that combining motor and cognitive measures can enhance prediction accuracy, replication and further investigation into the optimal combination of measures are needed. Notwithstanding this, the present study provides a proof of concept and underscores the potential benefits of a multidomain approach towards improving the prediction of amyloid burden. Our finding that the combined cognitive-motor model did not show better prediction capacity than the cognitive-only model for hippocampal volume, despite growing evidence highlighting the role of the hippocampus in motor learning,^34,35^ could possibly be related to the relatively simple nature of the motor sequence in our task. While the length of 4 elements was chosen deliberately to facilitate observable learning in the AD dementia group and minimize the risk of a floor effect, this approach may have inadvertently induced a ceiling effect. Future studies could consider more complex motor sequences (e.g., with more items) that are expected to yield a more gradual learning curve, to evaluate whether motor learning measures could be a valuable addition to standard cognitive assessments in predicting loss of hippocampal volume.

There are a number of limitations worth mentioning. First, the current sample comprised individuals classified as CNI, aMCI, or AD dementia, as initially diagnosed in a clinical setting and then confirmed by cognitive assessments and established protocols following ADNI criteria. However, we acknowledge that with recent scientific advancements and the availability of neuroimaging and blood-based biomarkers, the ability to determine etiology and therewith more precisely classify individuals has improved. A post hoc examination of amyloid status in our sample revealed that 25 of 28 AD dementia participants, 24 of 33 aMCI participants, and 8 of 52 CNI participants were amyloid positive. This predominance of amyloid positivity in the aMCI and AD dementia groups supports the effectiveness of ADNI criteria in accurately classifying these populations. Furthermore, the presence of amyloid in a subset of CNI participants aligns with prior research indicating that amyloid pathology can occur in cognitively healthy individuals.^42,43^ While ADNI criteria offer a reliable framework for classification, the challenge of definitively identifying the underlying pathology persists, underscoring the importance of integrating emerging biomarker techniques for improved diagnostic precision in future studies. Second, while the current study included three different widely-used AD biomarkers (i.e., amyloid burden, hippocampal volume, and *APOE* ɛ4 alleles), there are other potentially relevant biomarkers that could be considered in future studies (e.g., tau or inflammation). Third, since the current study focused on AD dementia and its biomarkers, these results provide little information about motor sequence learning in other types of dementia (e.g., vascular dementia, frontotemporal dementia, dementia with Lewy bodies). Fourth, as the sample sizes of the aMCI and AD dementia groups were relatively small, the generalizability of these findings to their respective populations remains uncertain. Relatedly, a limitation of running biomarker analyses across the full sample is that it is therefore possible that the observed associations between motor measures and AD biomarkers may be partly driven by between-group differences. Finally, future studies could consider using mouse tracking rather than key press responses, as this will allow researchers to differentiate between effects of aMCI/AD dementia on the *planning* of movements (i.e., a cognitive process) and the *execution* of movements (i.e., a process that is predominantly motoric in nature, though it can also comprise some cognitive aspects such as monitoring).

In summary, the present study found that despite well-established differences in general motor proficiency between CNI and aMCI/AD dementia individuals, motor learning abilities for short, deterministic sequences seem relatively preserved. However, aMCI and AD dementia were linked to poorer initial learning rates, which might be especially relevant to consider in relatively easy motor learning tasks where participants may reach peak performance early in the task. Although its observed independence of existing biomarkers indicates that sequence learning may not be suitable as an early disease marker by itself, the present statistical model comparisons suggested that adding motor measures to the standard neuropsychological assessment may enhance the prediction of amyloid burden. As such, future studies should continue to investigate the value of adding motor measures to the assessment and tracking of aMCI and AD, as well as considering motor functioning as an outcome of clinical trials in AD.

## Supplemental Material

sj-docx-1-alz-10.1177_13872877261485584 - Supplemental material for Motor sequence learning and learning to learn across the Alzheimer's disease continuum: Associations with biomarkers Supplemental material, sj-docx-1-alz-10.1177_13872877261485584 for Motor sequence learning and learning to learn across the Alzheimer's disease continuum: Associations with biomarkers by Marit F. L. Ruitenberg, Paula D. Carmona Osorio, Jace B. King, John M. Hoffman, Kevin Duff and Vincent Koppelmans in Journal of Alzheimer's Disease

## Data Availability

The data that support the findings of this study are not publicly available due to information that could compromise the privacy of research participants but are available from the corresponding author (M.R.) upon reasonable request.
