# Supplementary material for: Motor sequence learning and learning to learn across the Alzheimer's disease continuum: Associations with biomarkers
Source: J Alzheimers Dis. 2026 Sep 8;114(1):119–30. doi: 10.1177/13872877261485584 (PMC13675579; doi:10.1177/13872877261485584)
Supplement: Supplementary file 1 [file sj-docx-1-alz-10.1177_13872877261485584.docx]

**Supplemental Material**

**Motor sequence learning and learning to learn across the Alzheimer’s disease continuum: Associations with biomarkers**

**Supplemental Figure 1.** Results of principal component analysis on the motor and cognitive measures.
